# Evaluating pharmacological models of high and low anxiety in sheep

**DOI:** 10.7717/peerj.1510

**Published:** 2015-12-14

**Authors:** Rebecca E. Doyle, Caroline Lee, David M. McGill, Michael Mendl

**Affiliations:** 1 Animal Welfare Science Centre, Faculty of Veterinary and Animal Sciences, The University of Melbourne, Parkville, Australia; 2 Graham Centre for Agricultural Innovation (NSW Department of Primary Industries and Charles Sturt University), Wagga Wagga, Australia; 3 Agriculture, CSIRO, Armidale, NSW, Australia; 4 School of Veterinary Science, University of Bristol, Langford, United Kingdom

**Keywords:** Affective state, Behaviour, Diazepam, mCPP, Welfare

## Abstract

New tests of animal affect and welfare require validation in subjects experiencing putatively different states. Pharmacological manipulations of affective state are advantageous because they can be administered in a standardised fashion, and the duration of their action can be established and tailored to suit the length of a particular test. To this end, the current study aimed to evaluate a pharmacological model of high and low anxiety in an important agricultural and laboratory species, the sheep. Thirty-five 8-month-old female sheep received either an intramuscular injection of the putatively anxiogenic drug 1-(m-chlorophenyl)piperazine (mCPP; 1 mg/kg; *n* = 12), an intravenous injection of the putatively anxiolytic drug diazepam (0.1 mg/kg; *n* = 12), or acted as a control (saline intramuscular injection *n* = 11). Thirty minutes after the treatments, sheep were individually exposed to a variety of tests assessing their general movement, performance in a ‘runway task’ (moving down a raceway for a food reward), response to startle, and behaviour in isolation. A test to assess feeding motivation was performed 2 days later following administration of the drugs to the same animals in the same manner. The mCPP sheep had poorer performance in the two runway tasks (6.8 and 7.7 × slower respectively than control group; *p* < 0.001), a greater startle response (1.4 vs. 0.6; *p* = 0.02), a higher level of movement during isolation (9.1 steps vs. 5.4; *p* < 0.001), and a lower feeding motivation (1.8 × slower; *p* < 0.001) than the control group, all of which act as indicators of anxiety. These results show that mCPP is an effective pharmacological model of high anxiety in sheep. Comparatively, the sheep treated with diazepam did not display any differences compared to the control sheep. Thus we suggest that mCPP is an effective treatment to validate future tests aimed at assessing anxiety in sheep, and that future studies should include other subtle indicators of positive affective states, as well as dosage studies, so conclusions on the efficacy of diazepam as a model of low anxiety can be drawn.

## Introduction

In order to evaluate the welfare of animals more effectively research has led to the development of new behavioural and cognitive tests aimed at providing insight into welfare states (e.g., Harding, Paul & Mendl, 2004; Statham et al., 2013). However, interpretation of these tests can be difficult, especially when it is not clear what state (e.g., positive or negative) the treatment under investigation has induced. For example, different treatments inducing apparently similar states (e.g., negative states) can lead to different responses in certain tests (Doyle et al., 2011; Verbeek, Ferguson & Lee, 2014a). This calls into question the validity of the tests, but also whether the experimental treatments really did induce the states that they were designed or assumed to induce. This latter issue is a key challenge in the validation of new tests of welfare state, and it would be helpful to have a set of treatments that are known to reliably induce particular states in a target species, so that these can be used to evaluate the validity of new tests. One potentially promising type of treatment is pharmacological manipulation (Forkman et al., 2007; Mendl et al., 2009), which presents the possibility of triggering clear affective states. Drugs can be administered in a standardised fashion and with appropriate controls (e.g., saline injections), and they have the advantage of remaining active for the duration of a test (assuming appropriate half-life). This is not necessarily the case when using an environmental manipulation that, for example, can be applied prior to but not during a test.

Examples of drugs being used to validate tests for affective state in animal welfare research are becoming more prevalent (Destrez et al., 2012; Donald et al., 2011; Doyle et al., 2011; Rygula, Papciak & Popik, 2014; Verbeek et al., 2014b). In some cases, the drugs are chosen because of their effects in human subjects. For example, studies have shown that the serotonin agonist 1-(m-chlorophenyl)piperazine (mCPP) has an anxiogenic effect in both clinically anxious and healthy humans (Benjamin, Greenberg & Murphy, 1996; Charney et al., 1987; Kahn et al., 1990). Similarly, rats exposed to mCPP consistently perform behaviours indicative of an anxiety-like state including increasing escape attempts when in the elevated plus maze (Jones, Duxon & King, 2002), a reduction of exploratory behaviour in a light-dark box (Bilkei-Gorzó, Gyertyán & Lévay, 1998), and increased startle responses (Bijlsma et al., 2010).

Diazepam (DZP), a GABA agonist, is associated with a reduction in fearfulness and anxiety in humans (Roy-Byrne, 2004). In rats the administration of diazepam decreased anxiety-like behaviours in the elevated plus maze and reduced fear associated with a novel environment (Cole & Rodgers, 1995). Anxiolytic effects of diazepam have also been reported in pigs (Andersen et al., 2000; Dantzer, 1976; Dantzer, 1977), dogs (Ibáñez & Anzola, 2009), and cattle, where it was demonstrated to reduce behaviours indicative of anxiety, fear and frustration (Sandem et al., 2006).

Sheep are used broadly in medical research, including highly invasive procedures and pharmacological trials (Holland et al., 2013; Yates et al., 2010), are farmed in extensive conditions and in large numbers globally (Dwyer, 2009), and are a useful model species for other production animals as they are easy to maintain and have well-developed cognitive abilities (Greiveldinger, Veissier & Boissy, 2011). Pharmacological models of high and low anxiety states in this species would thus be of use in a range of research areas. Recent research has demonstrated that both mCPP and DZP may have similar effects in sheep to those observed in humans and rodents, but the results have been mixed (Destrez et al., 2012; Drake, 2006; Lee et al., 2009; Table 1). In an effort to evaluate these drugs as effective inducers of anxious (mCPP) and calm (DZP) states in sheep, the current study aimed to measure behavioural and cognitive differences in sheep following the administration of both drugs. Assessing both drugs in the same experiment allowed us to study their ability to generate states of increased and decreased anxiety relative to a control group, using the same tests and controlling for gender, age, experiential and environmental effects.

**Table 1:** Behavioural results from mCPP and Diazepam studies on sheep.

Drug	Reference	Dose (mg/kg)	Administration method	Drug-test interval	Animal numbers	Test	Finding
**mCPP**	Lee et al. (2009)	2	IM	30 min	32	Isolation box test (agitation scores only)	Increased agitation
						Spatial maze task—learning	No effect
						Spatial maze task—memory	No effect
	Drake (2006)	0.5	IM	20 min	5	Arena test	No effect
						Isolation box test (agitation scores and vocalisations)	No effect
		1			5	Arena test	Increased distance from human, zones crossed and vocalisations made
						Isolation box test (agitation scores and vocalisations)	No effect
		2			5	Arena test	No effect
						Isolation box test (agitation scores and vocalisations)	No effect
**DZP**	Drake (2006)	0.3	IM	20 min	5	Arena test	No effect
						Isolation box test (agitation scores and vocalisations)	No effect
		0.6		20 min	5	Arena test	No effect
						Isolation box test (agitation scores and vocalisations)	No effect
		0.9		20 min	5	General behaviour	Ataxia, reduced locomotion
	Destrez et al. (2012)	0.1	IV	10 min	10	Arena test	Less vigilant, but no effect on vocalisations, feeding or locomotion
						Suddenness test	Faster return to eating, but no feeding differences before the test
		0.1		10 min	8	Judgement bias test	No effect
				3 h		Judgement bias test	Reduced latency to approach one ambiguous location

Previous studies in sheep have proposed that evaluating simple cognitive processes can give insight into their affective states, with negative states reducing performance in simple tasks and, as in humans and rodents, increasing reactivity to startling stimuli (Greiveldinger, Veissier & Boissy, 2007; Greiveldinger, Veissier & Boissy, 2009). Similarly, increased activity and vocalisations are often observed in situations that sheep are motivated to avoid, and so increased activity in isolation can be reflective of higher levels of anxiety and distress in sheep (Beausoleil et al., 2012). Based on the literature, our *a priori* hypothesis was that sheep treated with mCPP would move more in isolation, have a reduced performance in a runway task (traversing a 150 m raceway to receive a feed reward), and have a greater startle response than control sheep, reflecting an increased level of anxiety. Conversely, it was hypothesised that sheep treated with diazepam would move less in isolation, have improved performance in the runway task and a smaller startle response than control sheep, reflecting a reduced level of anxiety (increased level of calmness).

## Methodology

The following protocol was approved by the Charles Sturt University Animal Care and Ethics Committee; protocol number 13/106

### Animals

Thirty-nine 8-month-old merino ewes averaging 30.2 kg (range 22.5–38.5 kg) were housed in groups of four to five in outside pens where they had *ad libitum* access to water and were fed a diet of Lucerne pellets, Lucerne hay and oats at a rate of maintenance plus 10%.

### Training of the runway task

The task was for sheep to walk down a 30 m laneway individually, and without visual contact with other sheep, to receive a feed reward. Training involved sheep moving from their home pen to the facility (150 m away) where they then had the opportunity to complete the task and receive the feed reward. Sheep were placed in a start box and given a maximum of 90 s to reach the feed reward and 30 s to consume the reward, which consisted of Lucerne hay and oats. If a sheep failed to reach the feed reward in the allocated 90 s, it was moved to the end of the laneway and then had the opportunity to eat. Sheep performed the task three times per day. For five consecutive days, sheep were trained to perform the task in groups of two to three sheep to facilitate them learning the task. After this sheep were considered to be habituated and were trained individually. Each sheep received individual training for seven days over a three week period. A regression analysis was performed to assess whether or not sheep had learnt the task, with four sheep failing to learn the task. Details on the training criteria are provided in the statistical analysis section.

### Treatments

Thirty-five sheep proceeded to the main trial and were randomly allocated to one of three treatment groups: control (*n* = 11), mCPP (*n* = 12) or DZP (*n* = 12). The mCPP group received an intramuscular injection of 1-(m-chlorophenyl)piperazine (Sigma-Aldrich, Castle Hill, Australia) at a rate of 1.0 mg/kg. The DZP group received an intravenous injection of diazepam (Provet, Sydney, Australia) at a rate of 0.1 mg/kg. Control sheep received a saline injection. Drug treatments were administered 30 min before testing in line with previous studies (Table 1).

Prior to the experiment, a dose of 2.0 mg/kg (Table 1) was tested on three sheep not involved in the experiment. The sheep displayed what the researchers perceived to be behaviours indicative of severe anxiety and discomfort including ataxic gait, head rolling and a stiff stance, none of which had previously been reported. This finding indicated that, from both an ethical and experimental perspective, the 2.0 mg/kg dose was too high and a dose of 1.0 mg/kg was used instead. When this dose was tested on another three sheep, also not involved in the experiment, the severe behaviours seen previously were not observed. At all dose rates, signs of mild distress had abated within 2 hs of initial administration.

### General movement and vocalisations

The time taken to move the sheep from a standard start point near the home pen to the start box of the runway task and the number of vocalisations made were recorded. This measured the effect of the drug on speed of movement before the test situation. No differences in gait were observed in any of the sheep.

### Runway task

Thirty minutes after the drug treatment, sheep were tested in the runway task. On the first entry to the start box, sheep were allowed to move down the length of the runway to receive a feed reward (‘runway task’), mimicking a normal training session.

### Runway startle task

On the second entry to the start box, a 5 s startle stimulus (rapid onset white noise at 98 dB (as measured 30 cm from the speaker; the closest distance that sheep could be)) was played as soon as the sheep left the box. The white noise was generated using the program Audacity^®^ 1.3.12 Freeware. The sheep were given 60 s to reach the feed reward.

Video recordings were taken of both tests and analysed by an observer blind to the treatments. The time each sheep took to reach the feed reward was recorded, as was the number of vocalisations. The level of ‘startle’ a sheep displayed to the audio recording in the startle test was subjectively scored using a 4-point scale (Table 2) which was adapted from the method reported by Greiveldinger, Veissier & Boissy (2007).

**Table 2:** Startle response scale.

Score	Description
0	No sign of startle
1	Stop movement
2	Stop movement and look towards the speaker
3	Display a visual contraction of the shoulders and/or hind quarters with flexion of the legs, stop movement, look towards the speaker

### Isolation test

At the end of the startle task, the sheep were moved to a 1 × 1 m pen with opaque sides for 1 min. The number of forward steps, the number of 180 ° turns, and the number of vocalisations that sheep made were scored from video recordings.

### Feed motivation test

Two days after the runway, startle and isolation tests, sheep were given the same drug or control treatment to test for feeding motivation. This was done independently of the other tests to assess the impact of the drugs on the appetite of the sheep. As the alpha half-life is 1 hr for DZP (Mandelli, Tognoni & Garattini, 1978) and 5.8 hr for mCPP (Gijsman et al., 1998), no traces of either drug would have been present in the sheep from the first trial.

Thirty minutes following treatment the sheep were tested for feeding motivation. Sheep were isolated in their home pen but retained close visual and auditory contact with conspecifics and were provided with approximately 150 g of oats in a shallow, transparent container. They were given a maximum of 1 min to eat the grain, and the time taken to commence feeding and the amount consumed were recorded. Exact weights of the feed provided to each sheep were recorded prior to the start of the test to ensure accurate consumption was recorded. Two sheep from the DZP group were removed from the test for unrelated illness before its commencement making DZP *n* = 10 for this test.

### Statistical analyses

All statistical analyses were performed in R version 3.2 (R Core Team, 2015).

#### Training criterion

To assess whether or not a sheep had ‘learnt’ the task, a regression analysis was performed for each sheep for each of the last three days of training. For each training day, the time required to reach the feed reward in each of the three training runs was used in a simple linear regression analysis. From the regression model, a predicted value for the final training run on that training day was calculated. Using this method, each sheep had a predicted ‘final training run’ time for each of the last three days of training. If any of the predicted run times for the last three training days was over 60s, that sheep was considered to have not ‘learnt’ the task. The time of 60 s was selected as this reflected the average response times of the sheep in the first two days of individual training without handler intervention.

The between-individual sheep variation accounted for approximately 30% of the total variation in the last three days of training (days five to seven). The regression analysis and 60 s cut-off time were used to account for a number of different ways sheep could have learned the task. For example, some sheep were generally faster during the training phase and learned the task quicker. In other cases, sheep may have been fast initially but then their latency times actually slowed, suggesting that they hadn’t necessarily learned the task, or were responding too inconsistently to be tested. Others were slow initially (i.e., greater than 60 s) but improved with time. This criterion covered numerous different situations where sheep may have been learning; thus providing a more robust approach to assessing learning than using an average time may lead to.

A regression was chosen as it captures trends in learning within and across days. Compared to taking an average of these training times, which gives a single value to compare to the cut-off time, the regression analyses carried out captures if an animal had improved or got worse over those final days. This approach is less susceptible to: (1) being skewed by a single poor performance or (2) missing a string of poor performances which indicate that an individual had not learned the task

#### Cox’s proportional hazards model

##### Runway task

Nineteen failed completions were recorded over both runway tasks, so these results were analysed with Cox’s proportional hazards model using survival analysis (Therneau, 2015; Therneau & Grambsch, 2000). Any sheep that failed to approach the feed reward within 60 s was deemed as a censored result, and this was recorded as a ‘survival’ incidence. Cox proportional hazards model considers explanatory variables that affect the hazard of an event happening. From the fitted model, the hazard ratio can be predicted to investigate the effect of a factor (drug treatment in this case) on whether or not a sheep was likely to approach the feed reward. Hazard ratio values are positive values ranging from zero to infinity. A hazard ratio of >1 indicates a higher likelihood of approaching the feed reward compared with the reference level for each categorical explanatory variable. Values between 0 and 1 indicate a lower likelihood of approaching the feed reward compared with the reference level. Note that the use of the term hazard in survival analysis does not necessarily imply a deleterious outcome and, in this study, the hazard refers to the sheep approaching the feed reward. Both the runway and startle runway tasks were analysed separately. Each of the drug treatments (control, DZP or mCPP) were fixed effects in the model and sheep was a random effect.

##### Feeding behaviour

A Cox’s proportional hazards model using survival analysis was also performed to investigate the effect of drug on feeding behaviour in the feeding motivation test and drug treatment was the only fixed effect. Similar to the runway task analysis described above, any sheep that failed to eat the feed reward within 60 s was deemed as a censored result, and this was recorded as a ‘survival’ incidence.

#### Other measures

Linear regressions were used to investigate differences in speed of movement of the sheep from a standard start point near the home pen to the start box of the runway task. Drug was the predictor variable in the model.

We recorded the number of vocalisations made during general movement, runway and startle runway tasks and the isolation test and during feeding motivation. General movement and both runway tests were variable in their duration, so vocalisations were calculated as vocalisations/min and analysed using a linear regression, whereas vocalisations in the isolation tests were analysed using a generalised linear model (GLM) with a Poisson distribution. Drug was the only predictor variable for these analyses. This same GLM method and model was also used to analyse steps and turns in the isolation test. There were no occurrences of sheep jumping in the isolation test. An ordinal logistic regression was performed on the scores for startle response, with drug as the fixed effect in the model.

## Results

### General movement and vocalisation

There were no statistical differences in the time taken to move sheep in each treatment group from the common start point to the start box of the runway task (*p* = 0.673), or for vocalisations (*p* = 0.466) during this time (Table 3).

**Table 3:** Results summary for the general movement test, runway task, runway startle task and isolation box test for each treatment group; standard error of the means presented in parentheses, different superscripts indicate statistically significant differences between treatments.

	General movement		Runway task	Runway startle task	Isolation box test		
Treatment	Average time (s)	Average vocalisations/min	Average vocalisations/min	Average vocalisations/min	Front steps	180° turns	Average vocalisations
Control	57 (3.1)	5.9 (1.3)	2.3 (1.1)^a^	1.7 (1.0)^a^	5.4 (0.9)^a^	3.3 (0.5)^a^	7.1 (2.8)
DZP	63 (5.7)	5.8 (1.1)	0.7 (1.1)^a^	1.8 (1.0)^a^	7.1 (0.8)^ab^	4.3 (0.8)^ab^	5.9 (1.0)
mCPP	61 (2.1)	6.4 (1.2)	7.5 (1.1)^b^	6.7 (1.0)^b^	9.1 (1.2)^b^	5.9 (1.3)^b^	5.0 (1.1)
*p*-val	0.673	0.932	<0.001	<0.001	0.0028	0.0105	0.467

### Runway task

**Figure 1:**
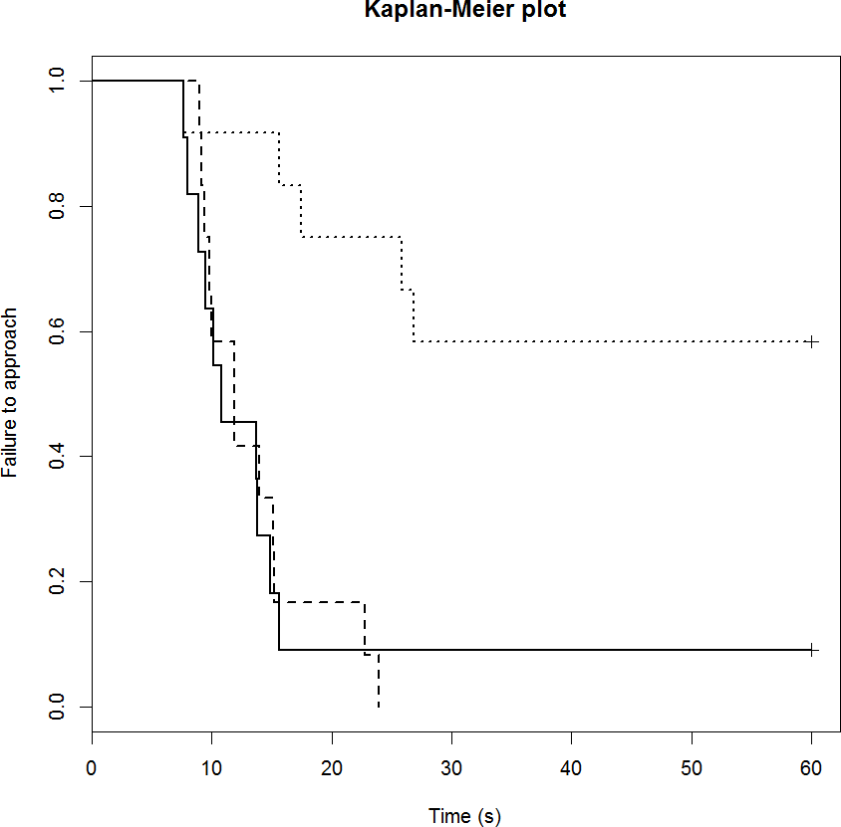
Kaplan-Meier curves for the runway task. Solid lines, control; long broken lines, DZP; dotted lines, mCPP. Every time a sheep approached the feed reward, the probability on the *Y* axis drops.

The drug treatment influenced sheep performance in the runway task, with the mCPP sheep being both significantly slower to complete the test and less likely to complete, compared to the two other treatment groups (Table 4). The mCPP treated sheep had a lower hazard ratio than the control group, indicating that they were less likely to complete the task. The DZP group had almost the same hazard ratio as the control group and were only slightly faster in their predicted approach time (Table 4; Time to approach). The Kaplan–Meier plot for this task (Fig. 1) shows the time of each approach and the proportion of sheep that failed to approach. The median approach time for the control group was 11 s and there was a 9% failure rate (1/11 animals did not complete the test). The median time was 12 s for the DZP group, and all sheep completed the test. The median of the mCPP group was 60 s as over half the animals did not complete the test (42% completed; 5 out of 12 sheep).

**Table 4:** Hazard ratio of sheep approaching the end of the test as affected by drug treatment, for both the known and startle runway tests.

Runway test	Drug	Coefficient^a^	SE (coeff)	Hazard ratio^b^	Time to approach (s)^c^	*P* value
Runway task	Control	Reference level				
	DZP	0.03	0.44	1.03 (0.6–1.47)	0.97×	0.94
	mCPP	−1.92	0.57	0.15 (0.1–0.19)	6.79×	<0.01
Runway startle task	Control	Reference level				
	DZP	0.5	0.45	1.64 (0.98–2.31)	0.61×	0.27
	mCPP	−2.04	0.68	0.13 (0.1–0.16)	7.7×	<0.01

**Notes.**

a Regression coefficient from the proportional hazards model.

b 95% CI in parentheses.

c Calculated as 1/hazard ratio (i.e., average time taken to reach the end of the test compared with the reference level).

### Startle runway task

**Figure 2:**
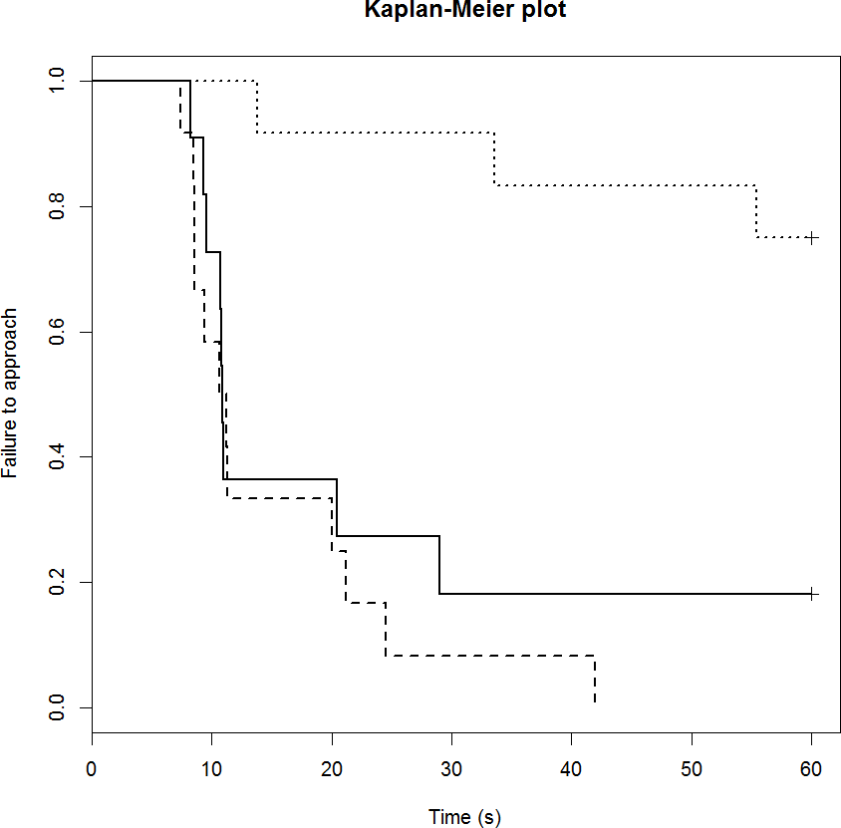
Kaplan-Meier curves for the runway startle task. Solid lines, control; long broken lines, DZP; dotted lines, mCPP. Every time a sheep approached the feed reward, the probability on the *Y* axis drops

The startle score for the mCPP group was significantly greater than the control group (1.4 vs. 0.6 respectively; *p* = 0.022), but there was no difference between the DZP (0.3) and control groups (*p* = 0.60). The drug treatment also influenced sheep performance in the runway component of the startle test in a similar way (Table 4). Only three out of 12 mCPP treated sheep completed the task (failure rate of 75%; Fig. 2). The control group had failure rate of 18% (2/11 sheep) and a median completion time of 11 s. The DZP treated sheep had a median completion time of 11 s and there were no failures.

Drug significantly affected vocalisations in each of the tests with the mCPP group vocalising significantly more than the other two treatments (Table 3; both *p* < 0.001).

### Isolation test

Sheep that received mCPP performed significantly more steps in the IBT test compared to either of the other treatments (*p* < 0.001; Table 3). There was no significant statistical difference in vocalisations.

### Feed motivation test

**Figure 3:**
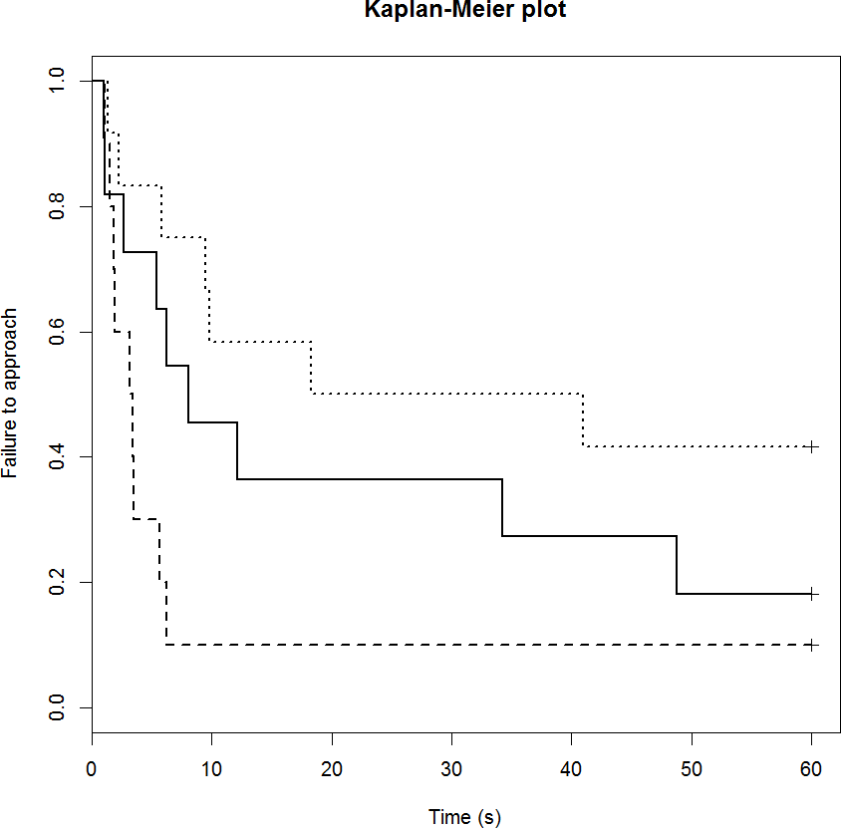
Kaplan-Meier curves for the feeding motivation test. Solid lines, control; long broken lines, DZP; dotted lines, mCPP. Every time a sheep commenced eating, the probability on the *Y* axis drops.

Neither drug treatment group differed in their feeding motivation compared to the control group (Table 5). The control group had failure rate of 18% (2/11 sheep) and the median time to eat was 8 s (Fig. 3). The DZP group had failure rate of 10% (1/10 sheep) and the median time to eat was 3 s. The mCPP group had failure rate of 42% (5/11 sheep) and the median time to eat was 30 s. Post-hoc least significant means analysis of the survival curves indicated a significant difference between the two drug groups (*p* < 0.05).

**Table 5:** Hazard ratio of sheep eating in the feed motivation test as affected by drug treatment.

Drug	Coefficient^a^	SE (coeff)	Hazard ratio^b^	Time to eat (s)^c^	*P* value
Control	Reference				
DZP	0.74	0.49	2.11 (1.31–2.9)	0.47×	0.13
mCPP	−0.59	0.51	0.55 (0.35–0.76)	1.8×	0.24

**Notes.**

a Regression coefficient from the proportional hazards model.

b 95% CI in parentheses.

c Calculated as 1/hazard ratio (i.e., average time taken to reach the end of the test compared with the reference level).

## Discussion

As hypothesised, sheep treated with mCPP displayed slower responses in the runway test, were more reactive to the startle test, vocalised more in both tests, and were more active in isolation, all indicating that they were more anxious than the control group. Thus, mCPP, at a dose rate of 1.0 mg/kg, is effective at inducing an anxious-like state in the sheep. In contrast to the hypothesis, no effects of DZP were identified. Both sets of results are discussed in detail below.

The mCPP treatment increased the level of activity in the isolation test, supporting previous results (Lee et al., 2009). Movement in restricted isolation has been clearly linked with agitation in sheep (Beausoleil et al., 2012; Bickell et al., 2011; Plush et al., 2011), and vocalisation is a measure of fear and distress during isolation (Cockram, 2004; Forkman et al., 2007). The higher amount of movement in the isolation test and the greater number of vocalisations in the runway task indicates that the mCPP treated sheep were more anxious than the other treatments.

The runway task also created a situation in which the sheep’s response to startle and subsequent recovery could be assessed, a method that has been used previously (Greiveldinger, Veissier & Boissy, 2007). In support of the hypothesis that mCPP treated sheep would be more anxious, they had a slower average completion time than the other two groups. Cognitive performance is optimal when an individual is experiencing mild stress or arousal (Mendl, 1999), however anxiety can distort risk assessment, disturb cognition and affect memory and attention (Lang, Davis & Öhman, 2000). Exposure to mCPP negatively affected cognitive performance of rhesus macaques in a variety of cognitive tasks assessed memory, attention, reaction time and motor coordination (Taffe et al., 2002). Studies in humans have also shown that mCPP affects perceptual and attentional tasks, but not those that use working memory (Sabbe et al., 2001; Silverstone et al., 1994). Together, these results support the findings of the current study, which uses a simple runway task to assess the impact of the drugs on task performance. The findings of Silverstone et al. (1994) that working memory was not affected by mCPP may help to explain why there was no difference in the spatial maze task previously used by Lee et al. (2004; Table 1) as this task requiring working memory. Further assessment of the specific type of cognitive function mCPP alters would allow further conclusions to be drawn.

The startle reflex is a ubiquitous, cross-species response to abrupt and intense stimulation, and the amplitude of the response varies according to the internal state of the organism, including its level of fear or anxiety (Grillon & Baas, 2003; Lang, Davis & Öhman, 2000) and is generally potentiated in negative states. Previously published work has demonstrated an increased startle response in rats following mCPP administration (Bijlsma et al., 2010; Risbrough & Geyer, 2005), indicating that mCPP potentiates startle responses and may hence induce anxiety and other negative states. In line with these findings, startle responses were potentiated in mCPP sheep relative to the control group, corroborating our proposal that mCPP induces an anxiety-like negative state in sheep.

When combined, these runway, startle and behavioural results from our study support mCPP as a pharmacological model of high anxiety in sheep; however, it is possible that these differences were the result of non-anxiety related changes (e.g., reduced feeding motivation, motor impairment). There was a risk that feeding motivation may be influenced by the pharmacological models, and therefore influence performance in the simple cognitive tasks. Indeed mCPP has been shown to suppress appetite and lead to reduced body weight in humans and rats (Lee et al., 2004; Sargent et al., 1997; Vickers et al., 2003; Wright & Rodgers, 2014). The independent feeding motivation test in our study did not detect differences between the mCPP and control groups, but there was a difference between the two drug treatments, suggesting an effect on appetite. Whether this is an effect of DZP, mCPP or small additive effects from both treatment groups is not clear however, as DZP can also increase appetite (Foltin, 2004; Gaskins, Massey & Ziccardi, 2008), although this same phenomenon is not always evident (Destrez et al., 2012; Sandem et al., 2006). Furthermore, the conclusions that can be drawn from the literature on the hypophagic effects of mCPP are limited as the studies listed above also reported behavioural changes that could be linked to general anxiety, and thus whether appetite is independent of general anxiety effects cannot be determined. Broadly, this highlights an ongoing issue of the effects that food-related tasks can have on the interpretation of results. These issues have been raised in other recently published studies investigating animal affect (Bateson et al., 2015; Verbeek et al., 2014b). It is noted however that gait was not affected by either drug, despite previously reported issues when using diazepam (Drake, 2006) and mCPP (Lee et al., 2004) in sheep. This is an important point as changes in movement are often used in behavioural tests designed to assess the affective states of domestic animals. Finally, it is noted that the number of animals in each treatment group in our study borders on what is considered to be the minimum recommended number of ‘events per variable’ for survival analysis (Peduzzi et al., 1995). Thus while a variety of the results indicate the validity of mCPP as a model of anxiety in sheep, we are cautious with the interpretation of these findings.

As no effects of DZP were evident, we cannot conclude that the treatment and dosage used generated a useful model of calmness/low anxiety in this study. However, it may be that the treatment was not effective during the test period. Destrez et al. (2012) identified reduced vigilance and a faster recovery from startle in sheep 10 min after DZP administration. Comparatively, we waited 30 min following administration before testing the sheep. Previous studies on the pharmacokinetics of DZP in sheep found that when administered intramuscularly, serum concentrations peaked 30 min after administration, and remained in circulation for over 5 hr (Moore et al., 1991). When considered in combination with the literature, it suggests that that the dose rate and administration-to-testing interval used in the current study were reasonable; however this cannot be verified, and as a result, the efficacy of DZP cannot be concluded on in this study. The limited pharmacokinetic information about these drugs raises another issue for other pharmacological treatments in future studies as many of the drugs currently used in affect-related animal research have not had comprehensive pharmacokinetic studies performed for the broad range of species we may want to test them in. This will be an important consideration if the use of drug models in applied ethology increases.

Whilst positive affective states are of growing interest in the assessment of animal welfare, methods for measuring them are limited (Yeates & Main, 2008). Thus a lack of appropriate measures to identify positive affective states in the tests used here may have prevented the detection of differences in the DZP group. Recent studies have shown that eye aperture, eye whites and ear postures may be useful ways to identify positive affective states in ruminants (Boissy et al., 2011; Proctor & Carder, 2015; Sandem et al., 2006), as well as there being evidence of qualitative behaviour assessment being effective (Rutherford et al., 2012), so measuring these in future studies using DZP could help clarify whether or not it is an effective model of low anxiety.

To conclude, there is good evidence that mCPP is an effective model of anxiety in sheep, and it negatively impacts on runway performance, enhances startle and increases distress during isolation; however, our results did not support DZP as an effective model of low anxiety in sheep. Based on these results, we suggest that mCPP is an effective treatment to validate future tests aimed at assessing anxiety in sheep. We also recommend that DZP is trialled in future studies that include other subtle indicators of positive affective states, and pharmacokinetic assessment, to provide a more rigorous examination of the effectiveness of it as a model of low anxiety in sheep.

## Supplemental Information

10.7717/peerj.1510/supp-1 Supplemental Information 1 Raw data

## Additional Information and Declarations

### Data Availability

The following information was supplied regarding data availability: Data have been uploaded under the ‘file’ tab.
